# Identification and transfer of spatial transcriptomics signatures for cancer diagnosis

**DOI:** 10.1186/s13058-019-1242-9

**Published:** 2020-01-13

**Authors:** Niyaz Yoosuf, José Fernández Navarro, Fredrik Salmén, Patrik L. Ståhl, Carsten O. Daub

**Affiliations:** 1 Department of Biosciences and Nutrition, Karolinska Institutet, 141 83 Huddinge, Sweden; 2 ​Science for Life Laboratory, Department of Gene Technology, KTH Royal Institute of Technology, Stockholm, Sweden; 3 Hubrecht Institute-KNAW (Royal Netherlands Academy of Arts and Sciences) and University Medical Center Utrecht, Cancer Genomics Netherlands, Utrecht, the Netherlands

**Keywords:** Spatial transcriptomics, Expression signature, Cancer diagnosis, Breast cancer, Machine learning

## Abstract

**Background:**

Distinguishing ductal carcinoma in situ (DCIS) from invasive ductal carcinoma (IDC) regions in clinical biopsies constitutes a diagnostic challenge. Spatial transcriptomics (ST) is an in situ capturing method, which allows quantification and visualization of transcriptomes in individual tissue sections. In the past, studies have shown that breast cancer samples can be used to study their transcriptomes with spatial resolution in individual tissue sections. Previously, supervised machine learning methods were used in clinical studies to predict the clinical outcomes for cancer types.

**Methods:**

We used four publicly available ST breast cancer datasets from breast tissue sections annotated by pathologists as non-malignant, DCIS, or IDC. We trained and tested a machine learning method (support vector machine) based on the expert annotation as well as based on automatic selection of cell types by their transcriptome profiles.

**Results:**

We identified expression signatures for expert annotated regions (non-malignant, DCIS, and IDC) and build machine learning models. Classification results for 798 expression signature transcripts showed high coincidence with the expert pathologist annotation for DCIS (100%) and IDC (96%). Extending our analysis to include all 25,179 expressed transcripts resulted in an accuracy of 99% for DCIS and 98% for IDC. Further, classification based on an automatically identified expression signature covering all ST spots of tissue sections resulted in prediction accuracy of 95% for DCIS and 91% for IDC.

**Conclusions:**

This concept study suggest that the ST signatures learned from expert selected breast cancer tissue sections can be used to identify breast cancer regions in whole tissue sections including regions not trained on. Furthermore, the identified expression signatures can classify cancer regions in tissue sections not used for training with high accuracy. Expert-generated but even automatically generated cancer signatures from ST data might be able to classify breast cancer regions and provide clinical decision support for pathologists in the future.

**Electronic supplementary material:**

The online version of this article (10.1186/s13058-019-1242-9) contains supplementary material, which is available to authorized users.

## Background

Breast cancer is the most common cancer and the highest incidence of all cancers in women with an incidence rate of over 1.6 million cases per year [1, 2]. The mortality rate is high over 90% when cancer cells spread systemically and colonize at distant organs from their tumors of origin [3]. Identification of both intra- and inter-tumor heterogeneity in breast cancer poses a significant challenge due to its genomic evolution that occurs during breast cancer progression. In depth characterization of the molecular heterogeneity is important to improve diagnosis, define prognostic biomarkers and for designing therapeutic strategies [4–6]. Understanding the cellular and molecular heterogeneity of tissue samples continues to be a challenge for high-throughput genomic technologies [7, 8]. Phenotypic markers are widely used to study cell heterogeneity with methods such as flow cytometry or immunohistochemistry [9, 10]. Bulk transcriptome analysis is used to study cell populations providing average expression levels for genes across large cell populations. However, these methods provide limited information about heterogeneous systems, including complex tissues consisting of various cell types or for cell types that are rare in a tissue. The molecular identities of diverse cells are lost during transcriptome analysis of bulk samples. Single-cell sequencing methods identify such subpopulations, which is important to study the intratumor heterogeneity that fosters tumor evolution. These methods need segregation of cells that disrupt the spatial context of cells from the tissue [11–16]. The physical location or coordinates of cells are important to understand tissue functionality and corresponding pathological changes. In the past, several methods have been developed to generate high-quality transcriptome maintaining the spatial information of cell localization [17–19].

Spatial transcriptomics (ST) is an in situ capturing technique, which uses a glass slide containing oligonucleotides to capture mRNAs, while maintaing the spatial information of histological tissue sections. Following cDNA synthesis, the resulting barcoded cDNA libraries are sequenced using standard RNA-seq technology [20]. Specific sequence barcodes allow to assign expression data to the positions on the slide. The efficiency of the method to capture transcriptomes from tissues with maintained positional information has been shown in several studies [21, 22]. In one of these studies, spatial gene expression profiles from breast cancer tissues were analyzed [20]. The role of the microenvironment in promoting tumor growth has proven important. The tumor microenvironment encompasses inflammatory cells, extracellular matrix, blood vessels, and stromal cells interacting with tumor cells for cancer growth and progression [23].

ST allows for a wide range of applications. The transcriptome is measured for the whole tissue section by sequencing that allows to compare different areas within that tissue section [20, 21]. For example, intratumor heterogeneity can be addressed by contrasting data from ST spots within one tumor or between different tumors [24]. Another application can be the identification of cancer subtypes and the simultaneous identification of the corresponding RNA biomarkers. Furthermore, known reference data such as genomic variation, for example SNPs from GWAS studies, can be cross-referenced against RNA biomarkers with the potential to assign possible functions for variants in poorly annotated genomic regions. In this concept study, we have applied computational machine learning algorithms to four ST datasets to characterize cancer regions in histological breast cancer tissue sections.

## Methods

### Data processing

For the study, we used four publicly available ST breast cancer datasets [20].

### Read alignment, annotation, and quantification

The ST sequencing results in paired-end reads. Read one (R1) contains the spatial barcode and the unique molecular identifier (UMI). Read two (R2) contains the transcript sequence information. All the datasets were processed using the ST Pipeline version 1.3.5 with default settings [20, 25]. The human genome hg38 and its corresponding annotation file were used for mapping and for assigning sequence reads to genes (annotation) [25]. The general statistics for the breast cancer datasets are shown in Additional file 1: Table S1.

The ST Pipeline generates a BED file containing the sequence reads mapped to genomic positions together with the spatial locations (the ST spots) of all the reads. All the transcripts annotated to the gene Malat1 were removed due to its overexpression and internal priming. We developed an open-source computational pipeline that uses the BED file to compute ST tag clusters (ST-TCs) for all the ST spots. Based on the transcription termination site (TTS) profiling, we computed TTS regions (ST-TCs) by peak calling of the transcripts genomic positions using the parametric clustering method paraclu [26], which was widely used for similar data in the FANTOM projects [26–28]. The ST-TC peaks and their corresponding expressions were visualized using the ZENBU interactive visualization tool [29]. The pipeline then computes the expression count matrices for each dataset, where the rows represent the ST spots (including X and Y coordinates on the ST slide) and the columns represent the ST-TCs with their expression values [20, 25].

We developed a Python open-source tool for unsupervised classification of ST spots based on the ST-TC expression profiles using the sklearn framework [30]. Datasets were filtered removing spots and ST-TCs with low expression (expressed in very few spots or very few ST-TCs, respectively). Expression count matrices were normalized with the size factors computed using DESeq2 [31]. A pseudocount of 1 was added prior to log2 transformation of the counts.

### Unsupervised classification of ST spots based on ST-TC expression patterns

Dimensionality reduction was performed using principal component analysis (PCA). PCA is a dimensionality reduction technique that simplifies the complexity in high-dimensional data (such as gene expression data) by transforming the data into fewer dimensions while retaining important trends and patterns [32]. Further, ST spots with close proximity in the principal component space were grouped into three groups (clustered with Ward2 method) based on euclidean distance. The clustered ST spots were plotted onto the hematoxylin and eosin (H&E)-stained images with specific colors representing the respective clusters. This grouping of the ST spots by the PCA does not take advantage of any expert knowledge of the histology samples and is therefore referred to as unsupervised classification.

### Manual identification of cancer subtype-specific ST spots

We classified (annotated) ST spots based on the morphology observed in H&E-stained histological tissue sections. The ST spots covering a minimum of 20 cells in the tissue section were selected. We identified subtype-specific ST-TCs (gene expression signatures) for non-malignant, ductal carcinoma in situ (DCIS), or invasive ductal carcinoma (IDC) regions (the three *classes* in machine learning terminology) using differential expression analysis (DESeq2, log2 fold-change > 2, false discovery rate < 0.01).

### Supervised classification of ST spots with machine learning

We used machine learning technique to learn expression signatures (train a model) for non-malignant, DCIS, or IDC regions. The regions were identified in two ways, by *supervised* expert annotation of the H&E images and independently in an *unsupervised* way by PCA. The identified expression signatures were used to characterize ST spots in ST experiments (testing of the model) which were withheld during model training.

For this, we developed a Python open-source tool (see references to tools) for supervised classification of ST spots using a multi-class support vector machine with the sklearn framework [30]. The tool requires one or multiple datasets for training and one dataset for testing. To assess the accuracy of the testing, the annotation of the test dataset needs to be known (so called *ground truth*) and provided as separate files. The training and test datasets are pre-processed and normalized as described in the unsupervised classification. The training datasets together with the ST spot labels were used to train a model using multi-class support vector machine (MC-SVM) method [33]. We chose default values for the hyper-parameters and used a linear kernel due to its simplicity with three classes. The trained model was then used to predict ST spot classes in the ST data (data not used in the training model) withheld during model training. The ST spots in the test dataset were plotted back onto the H&E-stained tissue image. The result of one test consists of associations of ST spots to classes. This association is given in terms of probabilities for each ST spot to each class. The probabilities might be interpreted as how much a cell type was represented in an ST spot in a mixed population of cells. The performance of the test result can be assessed by comparing to the known results using the F1-score, which is calculated based on true positive (TP) and true negative (TN) results. The F1-score ranges from 1.0 for perfect prediction to zero and can also be expressed in percent.

## Results

### Spatial transcriptomics datasets and gene annotation

The spatial transcriptomics (ST) technology places histological tissue sections on ST glass slides composed of 1007 ST spots covering the slide. Following tissue permeabilization, polyadenylated transcripts are captured on the slide and 3′ end sequencing libraries are produced containing spatial barcodes, determining where on the slide each transcript was captured [20].

Here, we developed a data processing and data analysis workflow for ST data, which extends the previously employed gene model centric expression analysis [20]. In brief, the 3’end sequencing reads were mapped to the reference genome and grouped into ST tag clusters (ST-TCs) using peak calling (Additional file 2: Figure S1) (see also “Methods” for more details). The resulting ST-TCs were annotated by associating them to nearby genes. In this way, more than one ST-TC might correspond to the same gene or an ST-TC might not be associated to any gene at all corresponding to non-annotated genomic regions. Data analysis was performed based on the ST-TCs independent of any gene model. The association of ST-TCs to genes was used for interpretation of results.

From four publicly available ST datasets of breast cancer tissue [20] processed together, we obtained a total of 979 ST spots covering the tissue samples, which corresponded to a total of 25,179 ST-TCs associated to 13,153 ENSEMBL genes. These ST-TCs corresponds to protein coding genes or non-protein coding genes or were not associated to any gene (non-annotated) (Additional file 1: Table S2). A total of 9369 ST-TCs were associated to exactly one gene, 3784 genes to more than one ST-TC, and 33 genes to 10 or more ST-TCs (Additional file 1: Table S3).

### Breast cancer expression signatures derived from expert annotated tissue sections

**Fig. 1:**
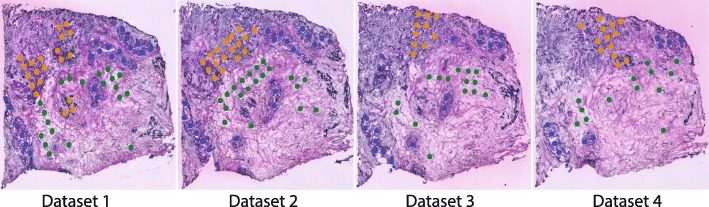
ST spots selected from four breast cancer histological tissue sections. ST spots selected from four contiguous histological sections from the same breast cancer samples with non-malignant (green), ductal carcinoma in situ (blue) and invasive ductal carcinoma (orange) regions

**Fig. 2:**
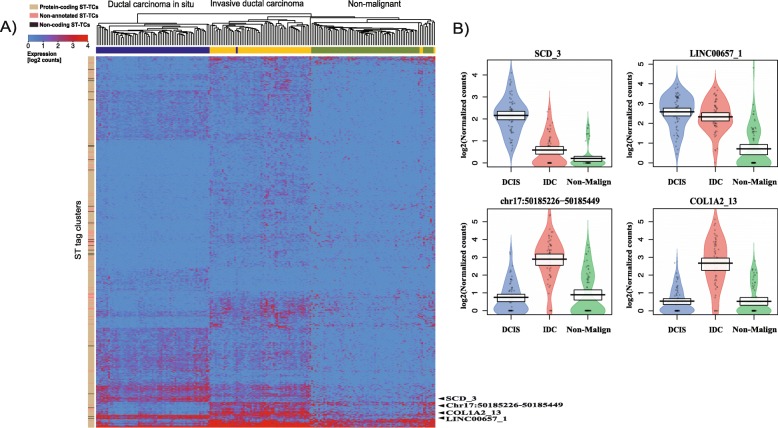
**a** Hierarchical clustering based on breast cancer expression profiles of differentially expressed 798 ST-TCs. The three group includes non-malignant, ductal carcinoma in situ and invasive ductal carcinoma regions. Columns are clustered by ST spots and rows are clustered by ST-TCs. **b** Examples of differentially expressed tag clusters among three breast cancer regions are shown in a pirate plot. The *Y*-axis is represented in log2 normalized counts

Hematoxylin and eosin (H&E)-stained histological tissue sections are routinely examined and classified by pathologists. We manually annotated the four breast cancer ST experiments and selected a total of 194 ST spots consisting of non-malignant regions, ductal carcinoma in situ (DCIS) regions, and invasive ductal carcinoma (IDC) regions (Fig. 1, Table 1A). These three regions are referred to as the *classes*. We conducted differential expression analysis (log2 fold-change > 2, FDR < 0.01, see “Methods”) to identify an expression signature of ST-TCs specifically expressed in any of these abovementioned three regions/classes. We obtained a total of 798 ST-TCs corresponding to 678 protein coding genes (696 ST-TCs), 23 non-coding genes (23 ST-TCs), and 79 non-annotated ST-TCs. This expression signature correctly classified 190 out of the 194 ST spots in the four available ST datasets (Fig. 2a).

**Table 1:** The number of ST spots from breast cancer tissue samples obtained by (A) manual annotation by pathologists and (B) automated annotation by PCA

	(A) Number of manually selected Breast cancer ST spots			(B) Number of automatically identified breast cancer ST spots		
Datasets	Non-malignant	DCIS	IDC	Non-malignant	DCIS	IDC
1	20	21	20	133	34	75
2	20	18	17	152	36	53
3	15	17	10	165	24	63
4	13	10	13	147	34	63
Sum	68	66	60	597	128	254

The differentially expressed transcripts can be regarded as marker signature for the three classes and in turn each class specifically expressed some of the marker transcripts (Fig. 2b, Additional file 3: Figure S2). For example, the analysis identified the non-coding gene LINC00657 as upregulated in DCIS and IDC regions compared to non-malignant regions (Fig. 2b). Several studies have identified the oncogenic role of LINC00657 by knockdown experiments, which significantly suppressed tumor cell growth and proliferation. The study showed its role in genome stability by inactivating an RNA binding protein that represses the stability and translation of mRNAs to which they bind [34–37]. We further performed gene ontology enrichment analysis of the gene signatures (798 transcripts). The analysis highlighted enrichment of gene sets for adherens junction (GO:0005912), cell-substrate junction (GO:0030055), anchoring junction (GO:0070161), and focal adhesion (GO:0005925) in all three analyses (Additional file 4: Figure S3). Adhesion complexes include adherens junctions, tight junctions, and gap junctions and are important for integration of signaling cascades. Disruption of these complexes might lead to impairment of normal tissue function and actuate pathophysiological disorders [38, 39].

### ST signature transfer of expert annotated breast cancer sections using a support vector machine

Machine learning has been frequently used for cancer prediction and prognosis. The method employs statistical, probabilistic, and optimization techniques to learn from known examples in order to recognize patterns in large complex datasets. Application examples range from general disease diagnosis to precision medicine [40] and include various clinical studies where outcomes were predicted for various cancer types and for cancer susceptibility [8, 41–43].

We first trained a machine to learn expression signatures based on ST-TC expression data for ST spots manually annotated to the classes non-malignant, DCIS, or IDC. For this, we used a multi-class (three classes) support vector machine (MC-SVM) which performs classification by constructing hyperplanes in a multidimensional space. Our workflow for the four breast cancer ST datasets used three datasets to train the machine (the machine generates a model) and the fourth dataset was used for validating or testing the model. This process of training on three datasets and validating on the fourth dataset was conducted four times in total and is often referred to as cross-validation technique [44]. The prediction accuracy of the model was assessed using the F1-score (see “Methods”) from the manually classified ST spots excluded for training. ST spots with unclear identity were not assigned to any class. The ST-TC expression data from the three training datasets for the same class were combined and then ST-TC expression signatures were identified in two ways: (i) 798 breast cancer signature ST-TCs differentially expressed between any of the three classes were used for model training, or (ii) all 25,179 ST-TCs were used for model training. The first signature can be regarded as a minimum signature containing only the most relevant ST-TCs for the signature.

### Selected breast cancer expression signature

**Fig. 3:**
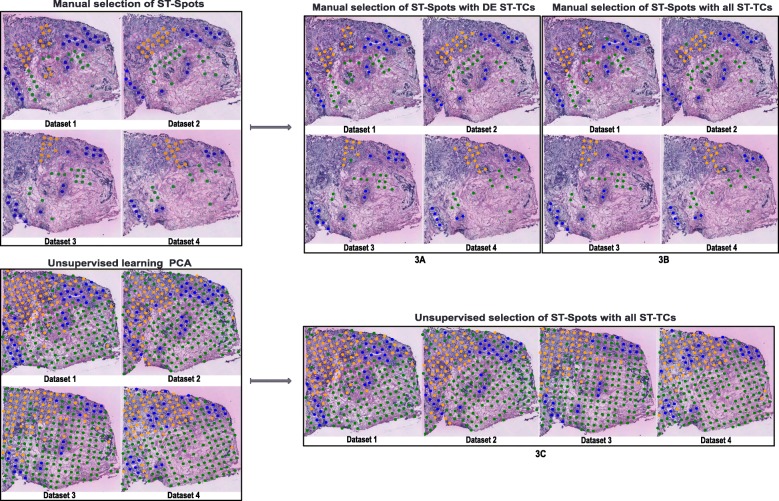
A, B, C: ST spots selected (manual (A, B) and automated selections (C)) from four histological tissue sections for training and testing are shown in the left box. The SVM model-predicted ST spots are shown in the right box. The spot colors represent non-malignant ST spots (green), ductal carcinoma in situ ST spots (blue), and invasive ductal carcinoma ST spots (orange)

From the manually selected ST spots of the four breast cancer datasets (Table 1A), we used 798 differentially expressed breast cancer signature ST-TCs to train the model. We selected 133 ST spots from the three breast cancer datasets 2, 3, and 4 (48 non-malignant spots, 45 DCIS spots, 40 IDC spots) to train the model. The model was then used to classify the ST spots of dataset 1 and to validate the accuracy of the prediction using the F1-score. The MC-SVM model classified the selected ST spots of the dataset 1 with an accuracy of 0.93, 1.00, and 0.92 for non-malignant, DCIS, and IDC spots, respectively (Fig. 3a, Table 2A). Three non-malignant ST spots were misclassified as IDC. We followed our workflow and conducted the same training/ testing cross-validation procedure for the remaining three datasets (train on three, test on the remaining) and obtained F1-scores in the range of 0.95–1.00 (Fig. 3a, Table 2A).

**Table 2:** Classification results (F1-scores) for testing ST-TC signatures with MC-SVM. The column “Dataset” indicates the sample on which the model was tested while the three remaining datasets were used for model training

	(A) Manually selected ST spots with DE ST-TCs			(B) Manually selected ST spots with all ST-TCs			(C) ST spots from unsupervised clustering with all ST-TCs		
Dataset	Non-mal.	DCIS	IDC	Non-mal.	DCIS	IDC	Non-mal.	DCIS	IDC
1	0.93	1.00	0.92	0.95	0.98	0.97	0.96	0.97	0.93
2	1.00	1.00	1.00	0.98	1.00	0.97	0.96	0.91	0.92
3	0.97	1.00	0.95	1.00	1.00	1.00	0.95	0.96	0.86
4	1.00	1.00	1.00	1.00	1.00	1.00	0.96	0.97	0.93
Avg	0.97	1.00	0.96	0.98	0.99	0.98	0.95	0.95	0.91

### All expressed transcripts without selection

We further tested the performance of the MC-SVM classifier for the same 133 ST spots without performing prior differential expression analysis. Here, we used all 25,179 ST-TCs for model training of the selected ST spots. The classifier identified ST spots in dataset 1 with an accuracy of 0.95, 0.98, and 0.97 for non-malignant, DCIS, and IDC spots, respectively (Fig. 3b, Table 2B). One DCIS and one IDC ST spot were misclassified. For the remaining datasets, the model classified the regions with F1-scores in the range of 0.97–1.00 (Fig. 3b, Table 2B). Compared to the model based on differentially expressed ST-TCs, this model based on all ST-TCs predicted the ST spots with slightly higher accuracy.

### Unsupervised identification of breast cancer signatures

**Fig. 4:**
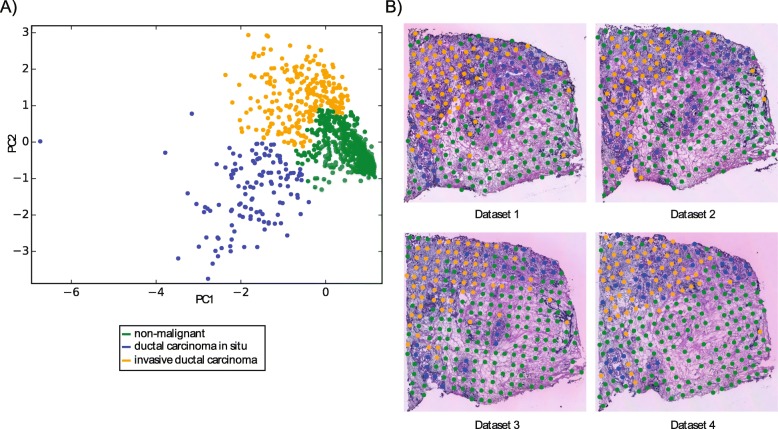
**a** The Principal component analysis and hierarchical clustering of 979 ST spots selected from four breast cancer tissue sections. **b** The clustered ST spots are plotted back to the corresponding histological tissue sections

We then evaluated whether the annotation of ST spots we performed manually could be performed by an unsupervised procedure in which no expert knowledge is provided and without selecting specific ST spots. For this, we combined all ST spots of all four available ST datasets (242, 241, 252, and 244 ST spots from datasets 1, 2, 3, and 4, respectively). We applied principal component analysis (PCA) on the data matrix consisting of 25,179 ST-TCs and 979 ST spots to place ST spots with similar expression close to each other in a 2-dimensional representation (the two first principal components) of the 25,179 dimensional expression space (Fig. 4a). To identify groups of ST spots in the PCA possibly corresponding to the three classes, we performed hierarchical clustering analysis (HCA) on the first two principal components and were able to identify three distinct groups of 979 ST spots (Table 1B, Fig. 4a) (see “Methods”). Given a number of expected clusters (*n* = 3 classes), HCA groups the ST spots on the 2-dimensional PCA plot such that each ST spot belongs to one cluster. Mapping these three groups of ST spots onto the four stained tissue images (Fig. 4b) revealed an overall accurate classification (186 of 194, 96%) with the ST spots for which expert annotation was available (Additional file 1: Table S4).

### ST signature transfer of automatically classified ST spots

We used the three groups of ST spots resulted from the unsupervised identification and performed the cross-validation workflow. The machine was trained on the combined three breast cancer datasets 2, 3, and 4 and validated on the dataset 1 resulting in an average F1-score of 0.94 (Fig. 3c, Table 2C). The same training and validation procedure was repeated for the remaining three datasets for which we obtained F1-scores in the range of 0.86–0.97 (Fig. 3c, Table 2C).

## Discussion

In this concept study, we have derived cancer expression signatures from spatial transcriptomics (ST) data, trained with one machine learning algorithm (MC-SVM) and evaluated the performance of the model to identify cancer regions. Four independent ST datasets were available and used in this study. Expert pathologist annotation of the H&E-stained tissue images provided classification of 194 ST spots of ductal carcinoma in situ (DCIS), invasive ductal carcinoma (IDC), and non-malignant tissue regions for all four experiments. We derived ST expression signatures for each of the three classes consisting of distinct sets of transcripts (798 ST-TCs) (Fig. 2a). In addition to protein coding gene-associated ST-TCs, ST-TCs for non-coding genes (23 out of 798, 2.9%), and non-annotated transcripts (79 out of 798, 9.9%) contributed to the expression signature. The extent of the contribution of non-coding transcripts was not evaluated in this study. The most differentially expressed non-coding or non-annotated TCs were predominantly expressed in DCIS and IDC regions emphasizing their potential role in cell proliferation and differentiation [36, 45–47]. The ST expression signature was able to classify ST spots with very high accuracy (190 out of 194, 97.9%) to non-malignant, DCIS, and IDC tissue regions.

In the past, various supervised machine learning algorithms were used in clinical studies to predict clinical outcomes based on expression signatures from bulk cancer samples, distinguishing DCIS from IDC or for extracting cell type-specific information from gene expression profiling from heterogeneous samples using deconvolution techniques [40, 41, 48–58]. These methods used a minimal set of differentially expressed and cell type-specific genes requiring specific analyses to obtain this subset. In this context, we continued with 194 ST spots from manual expert annotation and assessed the classification ability of a support vector machine (MC-SVM) to learn the three annotated classes based on the expression signature (small set of 798 transcripts/ ST-TCs) as well as on the complete set of all transcripts (ST-TCs). Classification results from both approaches gave comparable results of 97.7% and 98.3%, respectively (Table 2A, B). These results overall demonstrated the power of ST-TCs to classify cancer regions in breast cancer tissue sections. The smaller ST-TCs signature set gave comparable results to using the full set of ST-TCs. Classification on a smaller number of selected features might appear advantageous in terms of reduced model complexity. On the other hand, inclusion of all features would simplify the overall workflow by removing the selection process and might make it applicable in more complex workflows with fewer steps. The model which used all ST-TCs can also take advantage of features of the expression profiles that are not among the signature features identified by the differential expression analysis of the ST-TCs.

Our further analysis employed unsupervised classification of ST spots for identifying cancer regions. The resulting three distinct classes corresponded to the three expert annotated regions. The classification performance of the machine learning model (93.7%) was less accurate compared to the expert selected ST spots (97.7% and 98.3%, Table 2). The larger number of ST spots used by the unsupervised classification (979 unsupervised) might add variances in the data and complicate the classification task. Interestingly, for DCIS, the overall prediction accuracy is higher compared to IDC (Table 2). IDC regions might be more heterogeneous and might possibly contain other cell types such as fibroblasts.

We envision that in the coming years we will see simplification, further standardization, and reduced pricing for the ST protocol leading to extensive ST sequencing of samples of various cancer types. Here, the automated classification might become a powerful tool to support clinical pathologists in identifying cancer signatures. Moreover, the routine expert annotation of tissue sections might be used by an expert system to improve cancer signatures with increasing amounts of available data as well as in parallel to identify cancer subtypes with improved resolution. While the dataset employed is comprised of 979 sequencing libraries from four breast cancer ST experiments and constitutes the largest available ST breast cancer dataset so far, inclusion of additional individuals and breast cancer samples might be required to arrive at a cancer classifier for clinical usability.

The ST datasets are composed of sequencing data and corresponding detailed morphology of the stained tissue slides. In this work, we focused on the application of machine learning methods to the sequencing data. Machine learning has very successfully been applied to the classification of image data. We see great potential for a strategy applying machine learning to ST image data for detecting cancer regions while at the same time using machine learning to maximize the power of a corresponding expression signature.

Breast cancer manifests with subtypes that have different treatment responses and clinical outcomes. Identifying tumor heterogeneity in breast cancer regions is crucial for determining specific disease states and for starting suitable treatments early. Our application of a standard machine learning method to ST data clearly distinguished healthy and diseased areas in the tissue and most importantly identified regions containing both DCIS and IDC regions. We believe that detailed characterization of these regions might give us an insight into gene expression changes during the progression of breast cancer. More ST datasets containing such transitions might allow us to obtain detailed expression signatures and possibly a more detailed understanding of breast cancer progression. A more fine-grained resolution of the ST spots will enable higher resolution and allow detection of the transition between DCIS and IDC. This might pave the way towards the identification of new biomarkers specific to disease subtypes and hence cancer therapies for more personalized medicine. Histology is an efficient, effective, and relatively inexpensive diagnosis for breast cancer. We see the potential that ST technology might become a clinically usable complement to histology as the clinical gold standard.

## Conclusions

We report the application of one machine learning method to spatial transcriptomics data for the detection of DCIS and IDC cancer regions in individual breast tissue sections. We envision that computer-guided detection of cancer regions in spatial transcriptomics data will in the near future provide a clinical decision support for pathologists.

## Supplementary information

Additional file 1: **Table S1.** Mapping statistics of four breast cancer datasets. Table S2. ST tag clusters and associated genes for four ST datasets together. Table S3. Number of ST tag clusters per gene for four ST datasets. Table S4. The characteristics of the unsupervised ST breast cancer signatures. ST-TCs with at least one count were considered.

Additional file 2: **Figure S1.** Data driven and gene model independent data processing mapped ST sequencing reads (A) are grouped into ST tag clusters (ST-TCs) by peak calling (B).

Additional file 3: **Figure S2.** Volcano plot representation of differentially expressed ST-TCs. The tag clusters. Expression profiles of a) Non-malignant versus DCIS, b) DCIS versus IDC, c) Non-malignant versus IDC. The x-axis represents log2 expression fold change and the y-axis represents log10 *p*-value. The pirate plot of normalized log expression values for the differentially expressed tag clusters highlighted (examples) in volcano plot.

Additional file 4: **Figure S3.** Top 25 Enriched GO terms represented in dot plot. The size of the dots represent the number of genes associated with the given GO term and the color of the dots represent the P-adjusted values.

## Data Availability

Peak calling and expression matrix generation. https://github.com/jfnavarro/st_ts ST Analysis Pipeline https://github.com/jfnavarro/st_analysis

## Abbreviations

DCIS: Ductal carcinoma in situ
HCA: Hierarchical clustering analysis
IDC: Invasive ductal carcinoma
MC-SVM: Multi-class support vector machine
PCA: Principal component analysis
ST: Spatial transcriptomics
ST-TCs: Spatial transcriptomics tag clusters
TTS: Transcription termination sites
