# Supplementary material for: Identification and transfer of spatial transcriptomics signatures for cancer diagnosis
Source: Breast Cancer Res. 2020 Jan 13;22:6. doi: 10.1186/s13058-019-1242-9 (PMC6958738; doi:10.1186/s13058-019-1242-9)
Supplement: Supplementary file 1 — Table S1. Mapping statistics of four breast cancer datasets. Table S2. ST tag clusters and associated genes for four ST datasets together. Table S3. Number of ST tag clusters per gene for four ST datasets. Table S4. The characteristics of the unsupervised ST breast cancer signatures. ST-TCs with at least one count were considered. [file 13058_2019_1242_MOESM1_ESM.pdf]

Additional file 1

Identification and transfer of Spatial Transcriptomics signatures for cancer diagnosis

Tables

Dataset	%Trimmed	%ncRNA	%Mapped	%De-multiplexed	%Annotated	#Genes	#Reads
1	30.59%	18.58%	67.6%	97.9%	92%	23933	2337984
2	38.16%	10.3%	69.2%	97.64%	94%	23523	2224597
3	35.49%	19.44%	69.58%	97.89%	94%	24497	2637623
4	34.48%	10.79%	75.15%	97.37%	97%	23647	2659015

Table S1 : Mapping statistics of four breast cancer datasets.

	Protein coding	Non-coding	Non-annotated	Total
ENSEMBL genes	11,251	1,902	-	13,153
ST tag clusters	17,137	2,457	5,585	25,179

Table S2: ST tag clusters and associated genes for four ST datasets together.

	1	2	3-5	6-10	>10
Number of ST tag cluster per gene	9,369	2,497	1,129	125	33

Table S3: Number of ST tag clusters per gene for four ST datasets.

	non-malignant	DCIS	IDC
ST-TCs	16,895	23,823	23,211
Genes associated to ST-TCs	10,262	12,830	12,708
Expert annotated ST spots	68	66	60
Correctly classified ST spots	67 (98%)	64 (97%)	55 (92%)

Table S4: The characteristics of the unsupervised ST breast cancer signatures. ST-TCs with at least one count were considered.
