# Supplementary figures and images for: Identification and transfer of spatial transcriptomics signatures for cancer diagnosis
Source: Breast Cancer Res. 2020 Jan 13;22:6. doi: 10.1186/s13058-019-1242-9 (PMC6958738; doi:10.1186/s13058-019-1242-9)

Figure S1

A

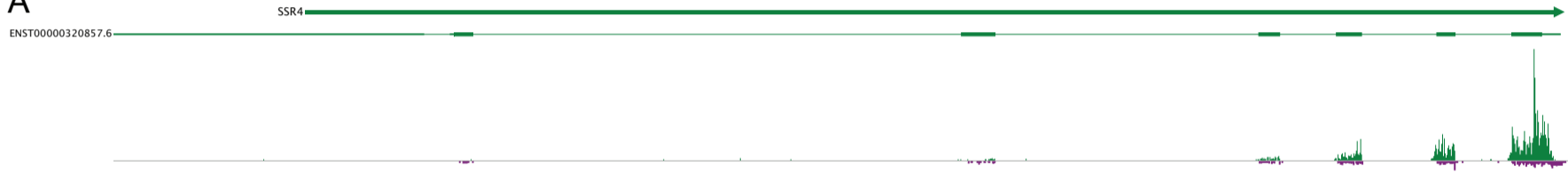

B

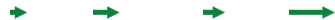

Supplement: Supplementary file 2 — Figure S1. Data driven and gene model independent data processing mapped ST sequencing reads (A) are grouped into ST tag clusters (ST-TCs) by peak calling (B). [file 13058_2019_1242_MOESM2_ESM.pdf]

Figure S2

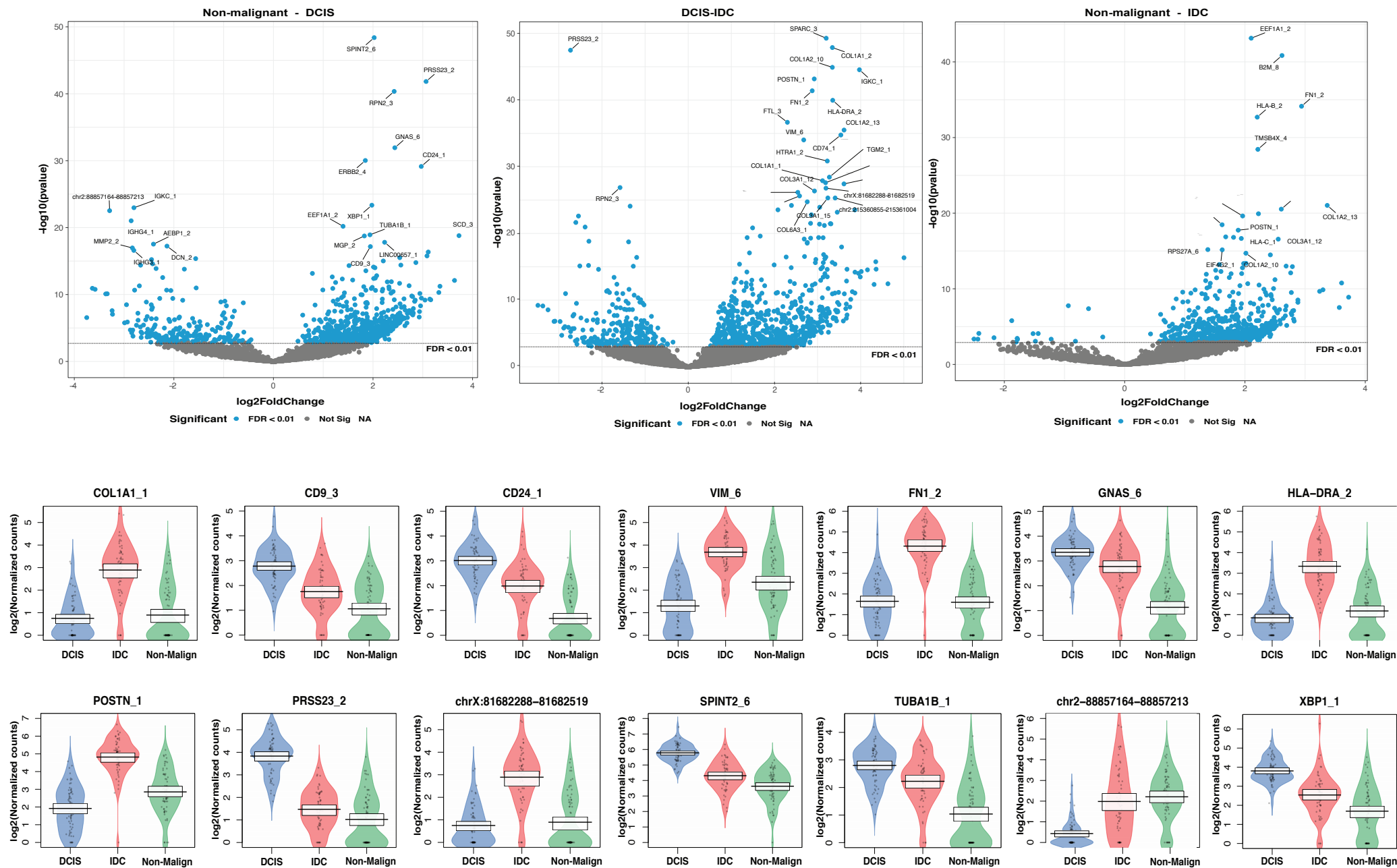

Supplement: Supplementary file 3 — Figure S2. Volcano plot representation of differentially expressed ST-TCs. The tag clusters. Expression profiles of a) Non-malignant versus DCIS, b) DCIS versus IDC, c) Non-malignant versus IDC. The x-axis represents log2 expression fold change and the y-axis represents log10 p-value. The pirate plot of normalized log expression values for the differentially expressed tag clusters highlighted (examples) in volcano plot. [file 13058_2019_1242_MOESM3_ESM.pdf]

Figure S3

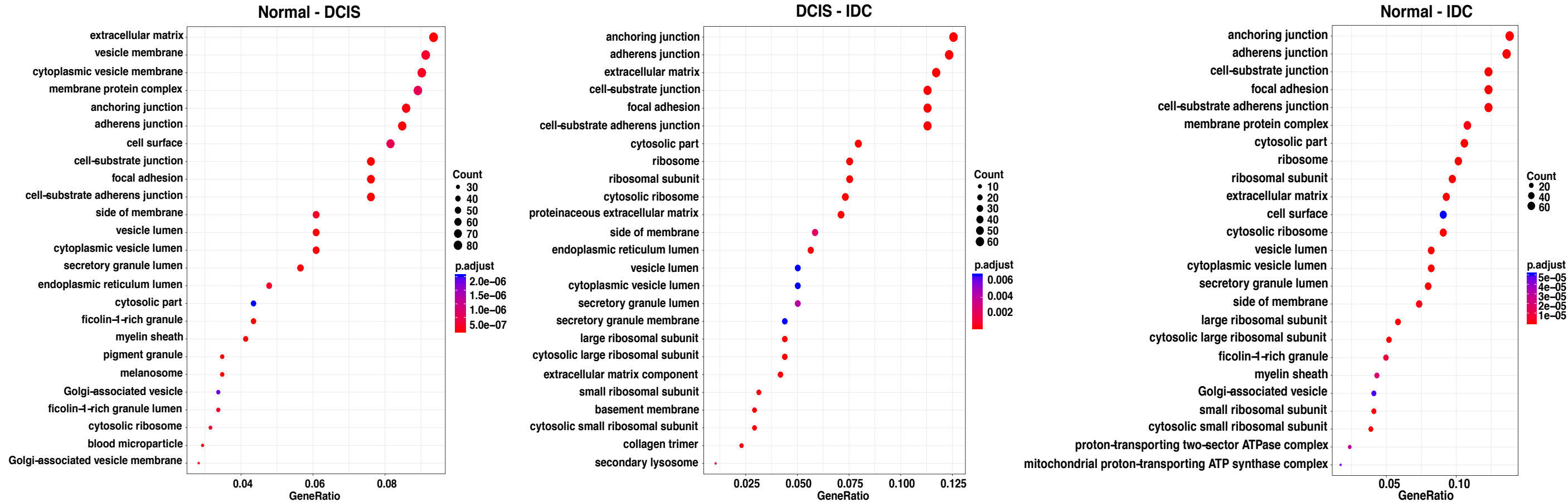

Supplement: Supplementary file 4 — Figure S3. Top 25 Enriched GO terms represented in dot plot. The size of the dots represent the number of genes associated with the given GO term and the color of the dots represent the P-adjusted values. [file 13058_2019_1242_MOESM4_ESM.pdf]
